# Cuproptosis-related gene index: A predictor for pancreatic cancer prognosis, immunotherapy efficacy, and chemosensitivity

**DOI:** 10.3389/fimmu.2022.978865

**Published:** 2022-08-25

**Authors:** Xufeng Huang, Shujing Zhou, János Tóth, András Hajdu

**Affiliations:** ^1^ Department of Data Science and Visualization, Faculty of Informatics, University of Debrecen, Debrecen, Hungary; ^2^ Faculty of Dentistry, University of Debrecen, Debrecen, Hungary; ^3^ Faculty of Medicine, University of Debrecen, Debrecen, Hungary

**Keywords:** cuproptosis, machine learning, pancreatic cancer, tumor microenvironment, immunotherapy, chemotherapy, gene signature

## Abstract

**Aim:**

The term “Cuproptosis” was coined to describe a novel type of cell death triggered by intracellular copper buildup that is fundamentally distinct from other recognized types such as autophagy, ferroptosis, and pyroptosis in recent days. As the underlying mechanism was newly identified, its potential connection to pancreatic adenocarcinoma (PAAD) is still an open issue.

**Methods:**

A set of machine learning algorithms was used to develop a Cuproptosis-related gene index (CRGI). Its immunological characteristics were studied by exploring its implications on the expression of the immunological checkpoints, prospective immunotherapy responses, etc. Moreover, the sensitivity to chemotherapeutic drugs was predicted. Unsupervised consensus clustering was performed to more precisely identify different CRGI-based molecular subtypes and investigate the immunotherapy and chemotherapy efficacy. The expression of *DLAT, LIPT1* and *LIAS* were also investigated, through real-time quantitative polymerase chain reaction (RT-qPCR), western blot, and immunofluorescence staining (IFS).

**Results:**

A novel CRGI was identified and validated. Additionally, correlation analysis revealed major changes in tumor immunology across the high- and low-CRGI groups. Through an in-depth study of each medication, it was determined that the predictive chemotherapeutic efficacy of 32 regularly used anticancer drugs differed between high- and low-CRGI groups. The results of the molecular subtyping provided more support for such theories. Expressional assays performed at transcriptomic and proteomic levels suggested that the aforementioned Cuproptosis-related genes might serve as reliable diagnostic biomarkers in PAAD.

**Significance:**

This is, to the best of our knowledge, the first study to examine prognostic prediction in PAAD from the standpoint of Cuproptosis. These findings may benefit future immunotherapy and chemotherapeutic therapies.

## Introduction

Copper is an indispensable element for human survival. However, its redox activity can be damaging to the cell that has evolved highly coordinated processes to chelate copper ions and transport them throughout the cell. Owing to its key involvement in pathways needed for normal cell development and metabolism, the copper level is typically dysregulated in malignancies (1). Hence, Cuproptosis as a unique cell death mechanism in which intracellular copper concentration plays a crucial role has attracted considerable interest in the scientific community (2).

The exploration of a potential connection between the copper equilibrium and the healthiness of the pancreas has a long history ever since the last century. As early as 1989, Dubick et al. found that nutritional copper deficiency raised a morphological change in the pancreas in female rats, and increased its susceptibility to oxidative damage (3). In 1997, Fields et al. reported that copper deficiency could lead to impaired functions and pancreatic atrophy in both male and female rats (4).

In the recent decade, increasing evidence suggested that abnormal buildup of copper stress might be linked to a lot of cancer types such as prostatic cancer (5), among which, pancreatic cancer was included. Clinically, according to the observation of Lener et. al., the concentration of copper ions was significantly elevated in patients with PAAD (6, 7).

Inspired by these findings, scientists started to develop new treatments attempting to regulate copper hemostasis. Existing studies suggested that copper-ionophores and copper-chelators might act as anticancer agents, although the lack of selectivity remained one of the most challenging obstacles in reality. Lately, breakthroughs in which the conjugation of targeting units with copper ionophores was proven to be effective occurred. Additionally, alternative options were brought forth by the exploitation of proionophores and the implementation of a nano-drug delivery system. One typical example in this field is the project led by Gaál et al. (8) who applied a thermosensitive liposomal formulation laden with copper and neocuproine to the C26 cancer cells in mice and detected both *in-vitro* and *in-vivo* toxicity.

Given that various types of cell death modalities (e.g. autophagy ferroptosis, etc.) were proven to be intimately associated with the eradication of tumors (9–11), we were inquisitive about the relationship between Cuproptosis and pancreatic adenocarcinoma (PAAD) for its exceptionally poor 5-year overall survival (OS) (12–20), and a striking fact that one of the Cuproptosis-related genes, *CDKN2A*, was a fully-investigated and well-known biomarker in PAAD at the same time.

Taking it altogether, in the present study, we optimized a Cuproptosis-gene index (CRGI) with important implications for prognosis, tumor immunology, molecular subtypes, and the efficacy of immunotherapy and chemotherapy through 10 mainstream algorithms in machine learning. Supplementarily, 3 essential CRGI genes (i.e., *DLAT, LIPT1*, and *LIAS*) were found with a promising potential to serve as diagnostic biomarkers through computational method analyzing open data combined with *in-vitro* validation.

**Figure 1:**
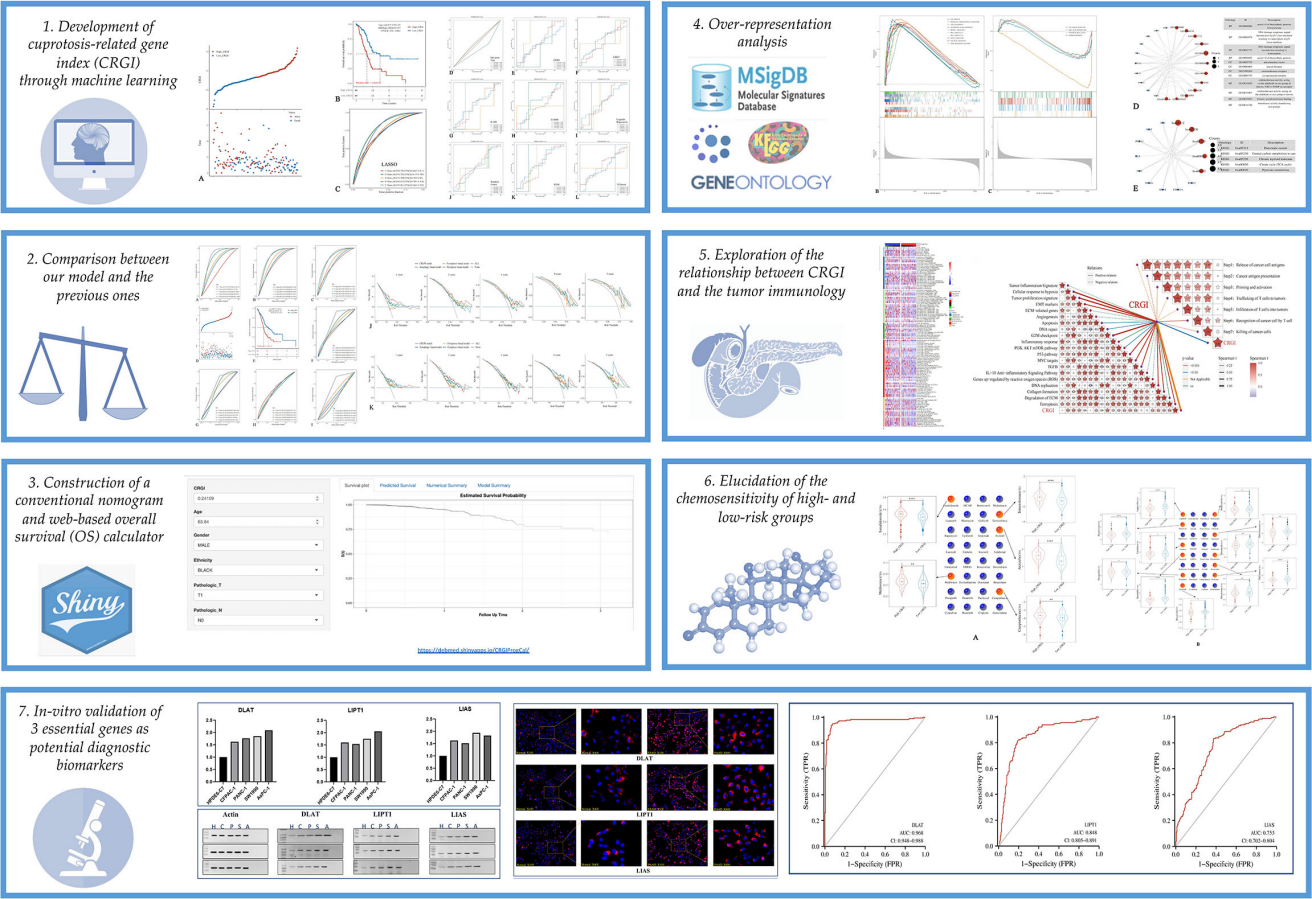
Graphical abstract of the present study.

**Figure 1** demonstrates the workflow of the present study briefly.

## Materials and methods

### Collection and integration of the transcriptome data and matched clinical information

In the present study, we retrospectively curated 10 Cuproptosis-related genes from the work of Tsvetkov et al. (2). The transcriptomic data and matching clinical information on pancreatic adenocarcinoma (PAAD) from publicly accessible sources, including the Cancer Genome Atlas (TCGA, https://www.cancer.gov/tcga, N = 177) and the Australian dataset of International Cancer Genome Consortium (ICGC, https://www.icgc-argo.org, N = 269) (21). All the data involved in the present study were processed by R Foundation (v 4.0.3) and corresponding R packages. Notably, if it wasn’t specifically mentioned, P-value<0.05 is considered statistically significant and might be annotated as * within the figures. Moreover, **, ***, and **** might appear within the figures to indicate the P-value thresholds 0.01, 0.001, and 0.0001, respectively. Besides, we took the academic writing style of Xie et al. (22) as a reference to construct the present manuscript.

### Machine learning in the development of cuproptosis-related gene index

10 mainstream machine learning algorithms were used in the optimization of CRGI, including least absolute shrinkage and selection operator (LASSO), decision tree, Gaussian mixture model (GMM), gradient boosted decision trees (GBDT), K-nearest neighbors (K-NN), light gradient boosting machine (LGBM), logistic regression, random forest, support vector machine (SVM), and extreme gradient boosting (XGboost) (23–31). Their performances were assessed by the time-dependent receiver operative characteristic (ROC) curves in which the area under the curve (AUC) represented the predictive power. The greater the AUC value indicated the better accuracy and robustness of the model. The ROC curve was created by the R package “timeROC”.

### Decision curve analysis

Usually, prognostic models and diagnostic tests are mathematically evaluated with measures of accuracy that do not consider clinical outcomes. To help improve such shortcomings, DCA was developed (32). It is often used to compare the efficacy of different predictive models and diagnostic tests to maximize the clinical benefits when false positives and false negatives are known to be unavoidable. In the present study, we performed this analysis by using the R package “ggDCA”.

### Construction of a conventional nomogram and corresponding calibration curve

The CRGI of our model that was optimized through the machine learning method as aforementioned was integrated as a prognostic indicator with other clinical factors to estimate the overall survival (OS) probability, through univariate and multivariate Cox regression, and a traditional nomogram with calibration curve was constructed from these results. The visualization was achieved by using the R packages “forestplot”, and “rms” (33, 34).

### Construction of an online OS calculator

By utilizing the analytic results acquired from the last step, we built an easy-to-use web-based OS calculator by the R package “DynNom” (35).

The calculator is available at https://debmed.shinyapps.io/CRGIProgCal/.

### Gene set enrichment analysis

The GSEA software (v 4.0.3, http://software.broadinstitute.org/gsea/index.jsp) combined with the gseKEGG and gseGO functions of the “clusterProfiler” package was used to investigate the underlying mechanism of the high- and low-CRGI groups, and further identified the KEGG and GO pathways that were significantly enriched through Gene Ontology (GO) and Kyoto Encyclopedia of Genes and Genomes (KEGG) databases (36–41).

### Estimation of the tumor microenvironment condition

The tumor microenvironment condition was assessed quantitatively by calculating the levels of stromal and immune cell infiltration using the expression profiles obtained from the TCGA dataset. This was done by the R package “ESTIMATE” in which the stromal score, immune score, and ESTIMATE score were calculated (42). The Wilcoxon t-test was performed for the calculation of each score to compare them in high- and low-CRGI groups.

### Screening of immune cell infiltration

The gene expression profiles were processed by integrating 7 mainstream immunoinformatic algorithms, including TIMER, CIBERSORT, CIBERSORT−ABS, QUANTISEQ, MCPCOUNTER, XCELL, and EPIC, and the immune cell infiltration matrix was obtained (43–45). The R package “ggplot2” was used to visualize the distribution of infiltration of diverse immune cell types as a heatmap.

### Cancer-immunity cycle and 19 known biological processes

In July 2013, Chen and Mellman (46) systemically described a series of self-sustaining stepwise events, termed the cancer-immunity cycle in which the anticancer immune responses eliminated the cancer cells efficiently. The cancer-immunity cycle consisted of 7 steps: “release of cancer antigens”, “cancer antigen presentation”, “priming and activation”, “trafficking of T cells to tumors”, “infiltration of T cells into tumors”, “recognition of cancer cells by T cells”, and “killing of cancer cells”.

As for the 19 known biological processes including tumor inflammation signature, cellular response to hypoxia, tumor proliferation signature, EMT markers, ECM-related genes, angiogenesis, apoptosis, DNA repair, etc., genes involved in this analysis were retrieved from the works of Wei et al. and Mariathasan et al. (47, 48).

### Prediction of the potential response to immune checkpoint blockade

The concept of immunotherapy for tumors was proposed at the end of the 19th century and refers to a treatment method that uses the body’s immune system to destroy cancer cells. The therapies that use immune checkpoint blockade have revolutionized the treatment of human cancer.

Herein, we firstly used the Tumor Immune Dysfunction and Exclusion (TIDE) algorithm which was developed as a computational method to model the primary mechanisms of tumor immune escape to predict the responsiveness of a single sample or a subtype based on expression profiling data (49). Since the original publication of the TIDE algorithm, researchers have widely applied it to their studies. A typical example could be seen in the work of Tang et al. in 2021 in the Journal of Translational Medicine (50). More similar studies could also be found elsewhere. For instance, the work of Chen et al. regarding a Necroptosis-related lncRNA signature in breast cancer also directly used the TIDE algorithm for predictive purposes (51).

Nevertheless, considering that the TIDE algorithm was merely experimentally verified in melanomas and non-small cell lung cancer, to further confirm the reliability of the TIDE prediction, the SubMap algorithm was implemented (52). It calculated the similarity of the expression profile of PAAD patients in the high- and low-CRGI groups to the urothelial bladder carcinoma (BLCA) patients recorded in the IMvigor210 dataset who responded or did not respond to the *PD-1* and *CTLA4* therapies, which in turn indirectly predicted immunotherapy efficacy (53). Such a solution was inspired by some previously published articles like the work of Yu et al. on lung adenocarcinoma (54).

### Unsupervised clustering through the K-means algorithm

The consistency analysis was performed using the R package “ConsensusClusterPlus (v 1.54.0)”, the maximal number of clusters was 6, and 80% of the samples were extracted 100 times through a re-sampling approach (55). The package generated the consensus matrix, empirical cumulative distribution function (CDF), and delta area plots for each selected K value.

Moreover, as a complementary confirmation, a principal component analysis (PCA) was conducted to elucidate if the samples were well-separated with the batch effect fully removed.

### Survival analysis

The Kaplan Meiler curve was applied to compare the survival difference between different groups. The P-value and hazard ratio (HR) with a 95% confidence interval (CI) were generated by log-rank test and univariate Cox proportional hazards regression. Both were done by the R package “survival” (56, 57).

### Evaluation of the response to chemotherapy

32 commonly used anticancer drugs were involved in the present study. Their half-maximal inhibitory concentration (IC50) values were predicted from the expression matrix by the pRRopheticPredict function of the R package “pRRophetic” (58).

### Cell lines and cell culture

There were 5 cell lines (i.e., normal human pancreatic ductal epithelial cell line: HPDE6-C7, and pancreatic cancer cell lines: CFPAC-1, PANC-1, SW1990, and AsPC-1) involved. HPDE6-C7 was cultured in a mixed solution of high-glucose DMEM and 10% FBS, CFPAC-1 was cultured in a mixed solution of DMEM/IMDM/1640 and 10% FBS, PANC-1 was cultured in a mixed solution of DMEM/1640 and 10% FBS, SW1990 was cultured in a mixed solution of DMEM/L-15/1640 and 10%FBS, and AsPC-1 was cultured in a mixed solution of DMEM/1640 and 10%FBS. All the aforementioned cell lines were incubated at 37°C and in a 5% CO2 incubator.

### Real-time polymerase chain reaction

The total RNA of cells was extracted through the one-step method (i.e., Trizol), then 2uL of which was used for reverse transcription. The quality of the cDNA was tested before further steps. The RT-qPCR was conducted under the following primer sequences design (**Supplementary Material T1**) and using the StepOne Software instrument (ABI, USA). The reaction conditions were 95°C for 5 min, 95°C for 10 s, 58°C for 20 s, and 72°C for 20 s. in total, 40 cycles were run. After the end of the reaction, the software automatically analyzed the fluorescence signal and converted it to the Ct value.

### Western blot

All the cell lines were washed by PBS 3 times and lysed by lyase, assisted with an ultrasonic cell crusher to ensure we could obtain the targeted proteins fully. The extracted proteins were quantified and loaded for SDS-PAGE gel for electrophoresis, transferred to PVDF membranes, and blocked by 10% milk. Later, the membranes were incubated with primary antibodies and secondary antibodies to form immunocomplexes which were visualized by enhanced chemiluminescence and followed by directly photographing and quantitative analysis.

### Immunofluorescence staining

HPDE6-C7 and AsPC-1 cell lines were used in the IFS validation and observed under 100- and 400-time magnification for the protein staining of *DLAT, LIPT1*, and *LIAS*, respectively. Information regarding the antibodies used in the present study is available in the **Supplementary Material T2**.

## Results

### CRGI was optimized from 10 mainstream algorithms

10 Cuproptosis-related genes were curated from the work of Tsvetkov et al. (2). Combined with 6 well-recognized biomarkers (i.e., *KRAS, TP53, SMAD4, BRCA1, BRCA2*, and *CDKN2A*) in pancreatic adenocarcinoma (PAAD), they were subjected to mainstream machine learning procedures to develop a Cuproptosis-related gene index (CRGI) (59).

**Figure 2:**
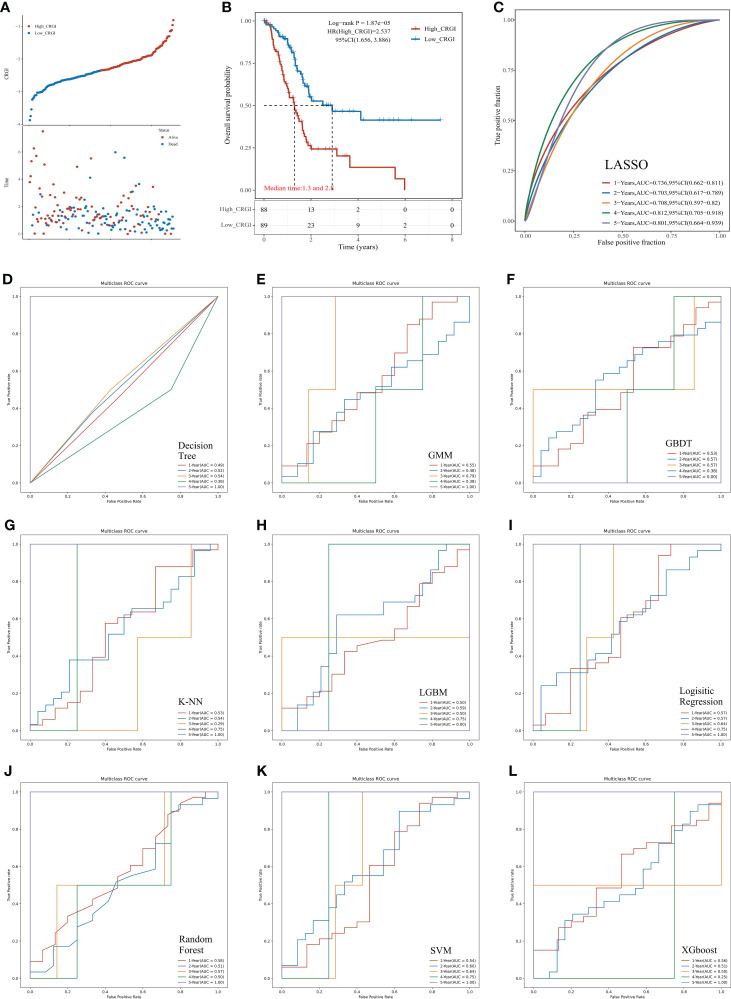
Predictive performance comparison of the 10 mainstream machine learning algorithms in the TCGA dataset. **(A)** Distribution of the CRGI and survival status of individual PAAD patients. **(B)** Survival analysis of the high- and low-CRGI groups. **(C)** The time-dependent ROC curve with the AUC value of 1-, 2-, 3-, 4-, and 5-year OS prediction of the best model optimized by LASSO penalized Cox regression. **(D–L)** The predictive accuracy of other machine learning algorithms (i.e., Decision Tree, GMM, GBDT, K-NN, LGBM, Logistic Regression, Random Forest, SVM, and XGboost, respectively).

In the TCGA dataset, among 10 mainstream machine learning algorithms, we optimized the best model through LASSO penalized Cox regression that had a leading AUC value in 1, 2, 3, and 4-year overall survival (OS) predictive performance, up to 0.736, 0.703, 0.708, 0.812, respectively (**Figure 2C**). The formula for the CRGI calculation is:



$$\begin{array}{l} CRGI\;=\;0.5316*KRAS\;+\;0.014*TP53\;–\;0.0407*CDKN2A\;–\;0.0999*SMAD4\\ \;+\;0.3768*BRCA1\;+0.0866*BRCA2\;–\;0.1292*LIAS\;–\;0.587*LIPT1\;–\;0.3158*DLD\;+\;0.4833*DLAT\;–\;0.3627*PDHA1\;\\ –0.3253*MTF1\;–\;0.1286*GLS \end{array}$$

**Figure 3:**
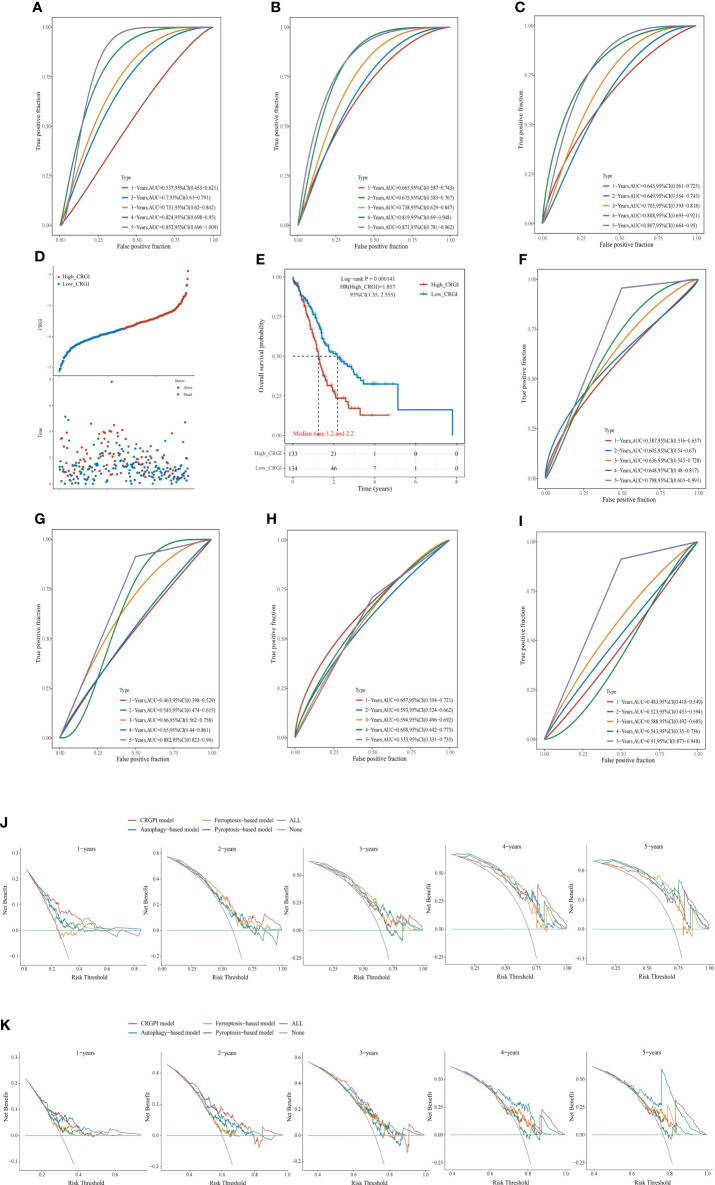
Comparison of other cell death mechanisms-based prognostic signatures in PAAD. **(A–C)** The ROC curve of autophagy-, ferroptosis-, and pyroptosis-based models in the TCGA dataset, respectively. Distribution of the CRGI and survival status of individual PAAD patients **(D)**, and Survival analysis **(E)** of the high- and low-CRGI groups in the ICGC dataset. **(F–I)** The ROC curve of our model, the autophagy-, ferroptosis-, and pyroptosis-based models in the ICGC dataset, respectively. DCA diagrams of our model, the autophagy-, ferroptosis-, and pyroptosis-based models in the TCGA dataset **(J)**, and ICGC dataset **(K)**. All: all positive; None: all negative. They are the extreme conditions that serve as background references.

Following the calculation of the CRGI, patients were separated into the high- and low-CRGI groups by the median value of all CRGI. It was observed that the number of patients who deceased significantly climbed up with the increase in CRGI value (**Figure 2A**). The survival analysis further revealed the fact that the low-CRGI group possessed a significant survival advantage (**Figure 2B**). Similar analytic results were found when we validated our model in the ICGC dataset (**Figures 3D, E**).

Interestingly, although the predictive performances of the other machine learning algorithms in the first 4 years were poor, 7 algorithms (i.e., Decision Tree, GMM, K-NN, Logistic Regression, Random Forest, SVM, and XGboost) possessed an AUC value = 1.000 in 5-year OS prediction (**Figures 2D–L**). Taking it altogether, we decided to choose LASSO as the final predictive model for the following comprehensive analytics.

### The predictive performance of CRGI was superior to that of the signatures derived from other cell death mechanisms

Recently, with the progressions made in the in-depth understanding of cell death mechanisms, a considerable number of prognosis-predictive gene signatures have been proposed. To clarify whether CRGI behaves better than those signatures originating from other underlying cell death mechanisms, we retrieved gene signatures proposed for PAAD that were derived from autophagy-, ferroptosis-, and pyroptosis-related genes (60–62). Then, we performed time-dependent ROC curves across the TCGA and ICGC datasets for each signature. In the TCGA dataset, our model possessed an AUC value of 0.736, 0.703, 0.708, 0.812, and 0.801 in the 1-, 2-, 3-, 4-, 5-year prediction (**Figure 2C**), whereas the autophagy-, ferroptosis-, and pyroptosis-based models possessed an AUC value of 0.537, 0.7, 0.731, 0.824, and 0.852; 0.665, 0.675, 0.738, 0.819, and 0.871; 0.643, 0.649, 0.705, 0.808, and 0.807 in the 1-, 2-, 3-, 4-, 5-year prediction, respectively (**Figures 3A–C**). A similar situation happened to the ICGC dataset where our model possessed an AUC value of 0.587, 0.605, 0.636, 0.648, and 0.789 in the 1-, 2-, 3-, 4-, 5-year prediction (**Figure 3F**). On the contrary, the autophagy-, ferroptosis-, and pyroptosis-based models possessed an AUC value of 0.463, 0.545, 0.66, 0.65, and 0.657, 0.593, 0.594, 0.608, and 0.483, 0.523, 0.588, 0.543, and 0.91 in the 1-, 2-, 3-, 4-, 5-year prediction, respectively (**Figures 3G–I**). Overall, comprehensively speaking, our model was deemed to possess the best AUC values, demonstrating the advanced stability and accuracy of CRGI.

Usually, it was considered rigorous enough to assess different models by comparing the AUC values of the ROC curve. However, as ROC analysis merely accounts for the specificity and sensitivity of the model, in the field of medicine, in case of unavoidable false positives and false negatives, one should maximize the clinical benefits from either result as possible. Therefore, we complementarily employed decision curve analysis (DCA) for each signature in the TCGA (**Figure 3J**) and ICGC datasets (**Figure 3K**). Within a DCA diagram, there were 2 baselines for reference purposes, annotated as “All” and “None” (i.e., All: all positive, None: all negative). The closer the curve of the corresponding model to them, the worse predictive performance in clinical practice indicated. Therefore, when the corresponding curve of a model possessed a higher position than others, it would mean that this model showcased a more practically useful prediction. Through the DCA diagrams, we found that although an exception exists in the 4- and 5-year curves in the ICGC dataset in which the curves of our model were less ideal than that of the autophagy-based model, the curve of our model was located superiorly to the others in the rest of the cases.

In fact, through the survival analysis in the TCGA (**Figure 2B**) and ICGC (**Figure 3E**) datasets, as aforementioned, less than 10% of the PAAD patients could survive more than 5 years (12–20). Hence, there were only 2 cases in the TCGA dataset and 1 case in the ICGC dataset who lived more than 4 years. The bias raised by this could probably be the reseason why our model performed similarly to the autophagy-based model in the 4- and 5-year prediction. Nevertheless, taking it altogether, we believed that our model had the strongest predictive power in general aspects.

### CRGI served as an independent indicator in PAAD prognostic prediction

**Figure 4:**
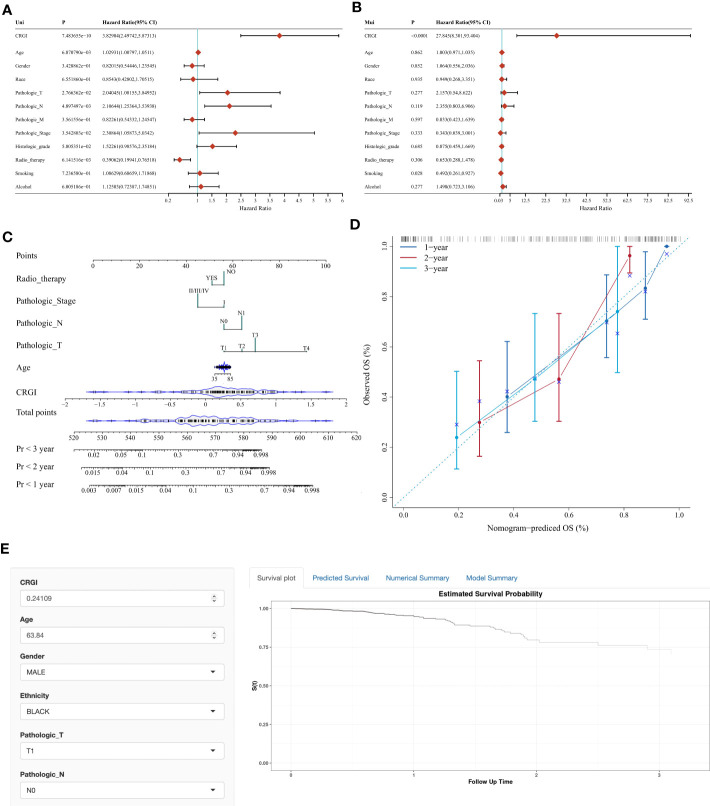
Nomogram with clinicopathological characteristics to predict OS in PAAD. **(A, B)** Forest plots of the results of the univariate and multivariate Cox regression, respectively. **(C, D)** Conventional nomogram and its calibration curve based on our model. **(E)** The screenshot of the web-based OS calculator calculating a fictional case.

Based on our model, we extracted the CRGI, age, gender, pathological status, TNM stages, histological grades, radiotherapy, smoking, and drinking information from the TCGA dataset, and carried out univariate Cox regression to examine if they are statistically correlated with prognosis and multivariate Cox regression to qualify their eligibility as independent prognostic indicators. It turned out that the CRGI, age, pathological T stage, pathological N stage, and radiotherapy were associated with prognosis as a result of the univariate Cox regression, while results of multivariate Cox regression furtherly indicated that the CRGI and smoking were independent prognostic indicators (**Figures 4A, B**).

According to these findings, we constructed a conventional nomogram (**Figure 4C**) that contained all the prognosis-related correlated factors (i.e., the CRGI, age, pathological T stage, pathological N stage, and radiotherapy) with a C-index up to 0.7883801. As generally, a C-index greater than 0.7 could be reckoned as a precise predictor, hence, it was thought that our model accurately predicted the prognosis of PAAD. To further test the robustness of its predictive results, we also compared them with real records in the TCGA dataset in a plot where a calibration curve indicated the deviation in a graphical manner (**Figure 4D**). As observed, the bias scale was acceptable and the trends of 1-, 2-, and 3-year prediction followed the ideal dash line relatively tight. As such, it was believed that the nomogram constructed in the present study exerted a satisfying performance.

To make it more user-friendly, the underlying statistics were implemented in a web-based OS calculator which assisted the clinicians to estimate the OS probability by entering the clinicopathological parameters, and the survival time of interest for prediction (**Figure 4E**). The calculator is available at https://debmed.shinyapps.io/CRGIProgCal/.

We also explored the correlation between the CRGI and different clinical factors (e.g., age, gender, tumor grading, etc.). It appeared that the CRGI was only associated with tumor grading, between G1 and G3, G2 and G3 (**Supplementary S1**).

### Over-representation analysis revealed the functional importance of CRGI in PAAD

**Figure 5:**
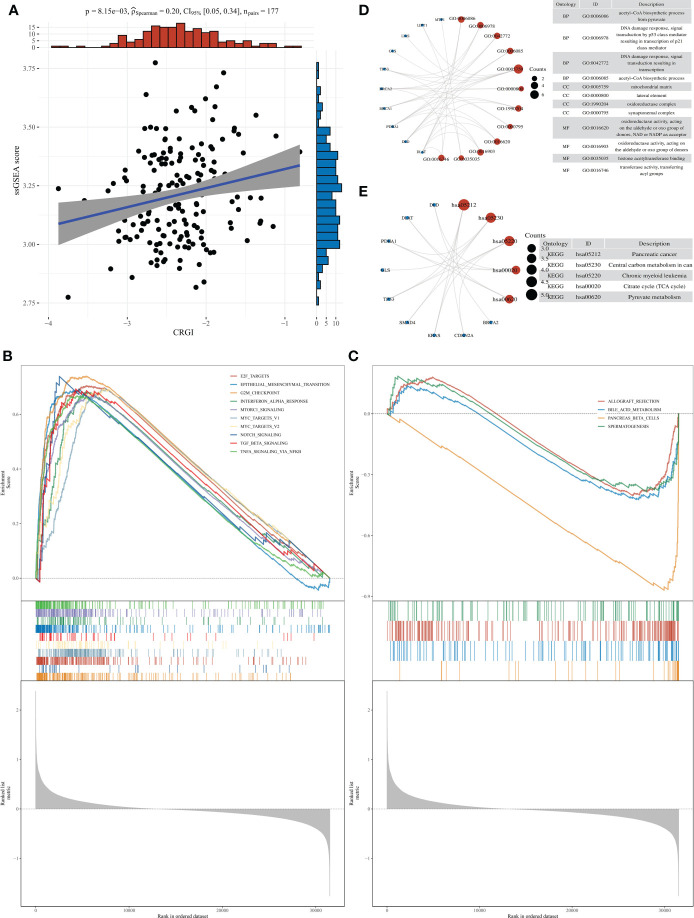
Over-representation analysis of CRGI in PAAD. **(A)** Correlation analysis of the CRGI and ssGSEA score. **(B)** The top 10 most enriched and upregulated cancer hallmarks in the high-CRGI group. **(C)** The 4 downregulated cancer hallmarks in the low-CRGI group. **(D)** The enriched GO terms of CRGI genes. **(E)** The enriched KEGG pathways of CRGI genes.

Over-representation analysis was conducted to unravel the functional mechanisms underlying the high- and low-CRGI groups through the ssGSEA. Results of the correlation analysis of the CRGI and the ssGSEA score implied that they were statistically significant (**Figure 5A**). Moreover, different cancer hallmarks were found enriched in the high- and low-CRGI groups with statistical significance. In total, 34 pathways were identified within the high-CRGI group. The most enriched pathways were upregulated and mostly related to cell proliferation, including G2M CHECKPOINT, E2F_TARGETS, etc. (**Figure 5B**). Within the low-CRGI group, 4 pathways were downregulated, mainly related to digestive functions, such as BILE_ACID_METABOLISM (**Figure 5C**).

We also performed GO terms and KEGG pathways enrichment analysis for the CRGI genes. Subsequently, enriched GO terms were found most relevant to the energy production within the mitochondria (**Figure 5D**), including the “acetyl-CoA biosynthetic process”, “mitochondrial matrix”, and “oxidoreductase complex”, etc. The other GO terms such as “DNA damage response” and “histone acetyltransferase binding” and so on were mainly related to the instability of the genome, which was reasonable as the majority of the CRGI genes were core genes involved in the copper-induced cell death mechanism (i.e., Cuproptosis) and the rest of the CRGI genes were driver mutation genes (i.e., *KRAS, TP53, SMAD4, BRCA1, BRCA2*, and *CDKN2A*) in PAAD. For the enriched KEGG pathways, the pathway entitled “pancreatic cancer” was found directly linked with the CRGI genes (**Figure 5E**). The other KEGG pathways like “TCA cycle” and “pyruvate metabolism” once again emphasized the critical role that the CRGI genes played in cellular physiology.

### CRGI was associated with the tumor microenvironment condition, cancer-immunity cycle, and immunotherapy efficacy

**Figure 6:**
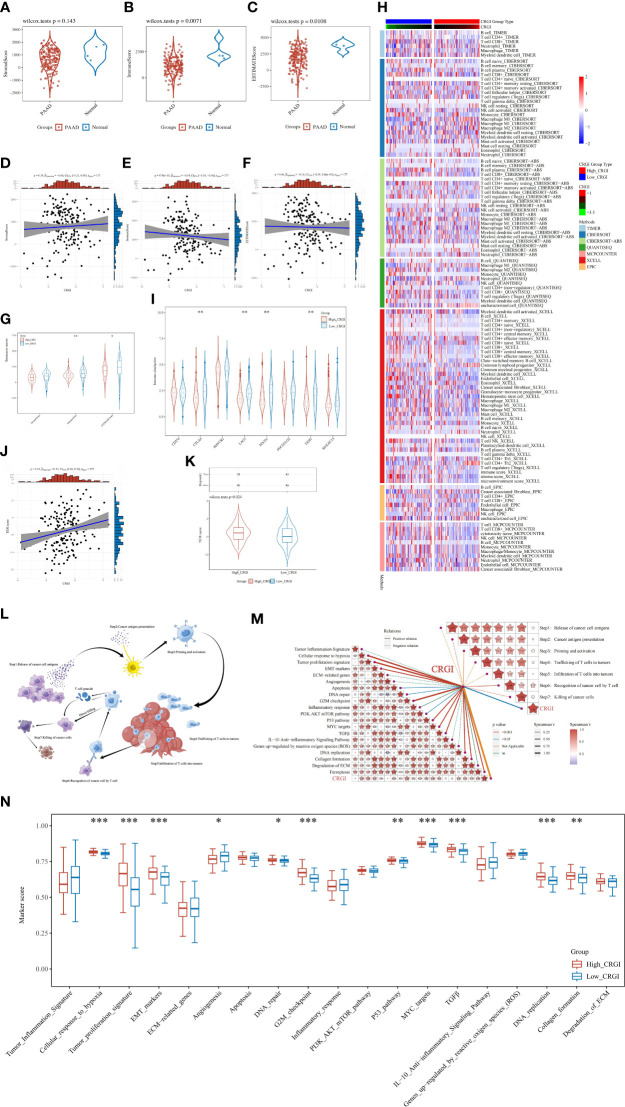
In-depth analytics on the relationship between CRGI and the tumor microenvironment condition, immune cell infiltration, immunotherapy efficacy, TMB, as well as the cancer-immunity cycle. **(A–C)** Comparison of the stromal, immune, and ESTIMATE scores of tumorous and normal tissues in the TCGA dataset. **(D–F)** Correlation analysis between the CRGI and the stromal, immune, and ESTIMATE scores. **(G)** The violent plot demonstrated the comparison of the stromal, immune, and ESTIMATE scores in the high- and low-CRGI groups. **(H)** The heatmap demonstrated the diverse immune cell types in the infiltration process. **(I)** The violent plot demonstrated the comparison of the expression of the representative genes of the soundest immune checkpoints in the high- and low-CRGI groups. **(J)** Correlation analysis between the TIDE score and CRGI. **(K)** The violent plot demonstrated the comparison of the TIDE scores in the high- and low-CRGI groups. **(L)** Graphical demonstration of the cancer-immunity cycle. **(M)** Correlation analysis between the CRGI and the main steps of the cancer-immunity cycle as well as the 19 known biological processes. **(N)** The boxplot demonstrated the comparison of marker scores in the high- and low-CRGI groups. *, **, ***, and **** indicate the P-value thresholds 0.05, 0.01, 0.001, and 0.0001, respectively.

It has been widely believed that cancers are essentially considered as dynamic ecosystems wherein subclone populations of most cancer cells and non-malignant cells in the tumor microenvironment engage cooperatively to promote the disease progression. Therefore, it is necessary to investigate the general appearance of the tumor microenvironment. Herein, by utilizing the clinical information curated from the TCGA cohort, we utilized the R package “ESTIMATE” to elucidate it in a quantitative way, through which we found that except for the stromal score, the immune score and ESTIMATE score were found statistically significant and that higher immune and ESTIMATE scores were observed in normal tissues than that of PAAD tumor tissues (**Figures 6A–C**). Then, we analyzed the correlation between the CRGI and stromal, immune, and ESTIMATE scores, respectively. It was found that the CRGI was negatively associated with the immune score with statistical significance (**Figures 6D–F**). We also analyzed the aforementioned scores in high- and low-CRGI groups, and it was observed that there was a difference in immune score and ESTIMATE score with statistical significance in which the low-CRGI group possessed a relatively higher immune score and ESTIMATE score (**Figure 6G**). These findings supported the idea that CRGI as a classifier of the high- and low-CRGI groups played an essential role in the distinguishment of the tumor microenvironment condition in PAAD. On the other hand, we exhaustively screened the immune cell types in the tumor infiltration process in the high- and low-CRGI groups by integrating 7 mainstream immunoinformatic algorithms, including TIMER, CIBERSORT, CIBERSORT−ABS, QUANTISEQ, MCPCOUNTER, XCELL, and EPIC. It was found that the immune cell types involved in this process were very much diverse. In detail, immune cell types such as B cells, CD4+ T cells, CD8+ T cells, regulatory T cells, myeloid dendritic cells, macrophages, NK cells, monocytes, and endothelial cells were found to dramatically differ from the high-CRGI group to the low-CRGI group (**Figure 6H**).

Immune checkpoints are negatively regulatory proteins in the immune system that are indispensable for maintaining self-tolerance, preventing autoimmune responses, and minimizing tissue damage. They function by controlling the timing and intensity of immune response. In immunotherapy, the overexpression of immune checkpoints inhibits the function of immune cells so that the body cannot produce an effective anti-tumorous immune response, which ultimately leads to immune escape. Therefore, to fully evaluate the potential impact on immunotherapy efficacy caused by CRGI, we also analyzed the difference in the expression of the representative genes of the soundest immune checkpoints in the high- and low-CRGI groups in the TCGA cohort, including *PD-1* (i.e., protein of *PDCD1* gene), *PD-L1* (i.e., protein of *CD274* gene), *PD-L2* (i.e., protein of *PDCD1LG2* gene), *CTLA4, HAVCR2, LAG3, TIGIT*, and *SIGLEC15*. It was found that there are statistical significances in the expression of *PD-1, CTLA4, TIGIT*, and *LAG3* (**Figure 6I**). It was also observed that there were higher expression levels in the low-CRGI groups, hindering that patients with low-CRGI might be benefited more from immunotherapy.

In addition, after integrating the clinical data curated from the TCGA cohort, through the TIDE algorithm (i.e., a scoring system that inversely reflects the immunotherapy efficacy), we predicted the potential response of PAAD patients to immune checkpoint blockade. To be more exact, we first conducted a correlation analysis in which we found that the TIDE score and the CRGI showed a significant correlation statistically with a P-value = 0.01 and a Spearman coefficient = 0.19 (**Figure 6J**). Then, we stepped forward, finding that in the high- and low-CRGI groups, there was also a distinct difference in which the low-CRGI groups demonstrated a prominently lower TIDE score than that of the high-CRGI group, implying that there were certain advantages in immunotherapy efficacy in the low-CRGI groups (**Figure 6K**).

As the TIDE algorithm was merely verified with real-world data in melanoma and non-small cell lung cancer, it is of nature to be concerned with the reliability of the predictive results. Therefore, to further confirm the reliability, we utilized the SubMap algorithm to validate the aforementioned results. We firstly screened the immunotherapy responders and non-responders using the CRGI as a classifier, finding that patients with lower CRGI in the IMvigor210 dataset were more responsive than those with higher CRGI (**Supplementary S2A, B**). However, as the SubMap algorithm was principally an inference-making machine purely based on the statistical similarity between the expression profiles, objective differences were inescapable, which might lead to deviations. Moreover, although it was the best option under the given conditions, the IMvigor210 dataset was somewhat less organized. Therefore, taking it altogether, the Bonferroni adjusted P-value was not ideal, despite a statistical similarity between the expression profiles of PAAD and BLCA could be seen (**Supplementary S2C**). Overall, the patients with low-CRGI in the IMvigor210 dataset demonstrated a certain positive association with the responsiveness of *PD-1* therapy, which once again implicated that they might be more suitable for immunotherapy.

As Cuproptosis is a cell death mechanism that may raise immune reactions, it is of great interest to investigate its potential underlying anticancer mechanisms in the tumor immunity aspect. Meanwhile, the cancer-immunity cycle proposed by Chen and Mellman (46) and the 19 known biological processes summarized by Wei et al. and Mariathasan et al. (47, 48) were hot research topics over the past decade, which were widely believed to be the key paths toward tumor malignancies. Hence, to boost the direct understanding of the bridge between the CRGI and the development of cancer, the correlation analysis between the CRGI and cancer–immunity cycle together with 19 known biological processes was performed based on the TCGA cohort (**Figure 6L**). As a result, the CRGI presented a significantly positive relationship with “cancer antigen presentation”, “recognition of cancer cells by T cells”, and “killing of cancer cells” in the cancer-immunity cycle, and “tumor proliferation signature”, “cellular response to hypoxia”, “EMT markers”, “apoptosis”, “DNA repair”, “DNA replication”, “G2M checkpoint”, “PI3K AKT mTOR pathway”, “MYC targets”, “P53 pathway”, “TGFβ”, “collagen formation”, and “ferroptosis” in the 19 known biological functions (**Figure 6M**). Similar differences with statistical significance also existed between the high- and low-CRGI groups, indicative of the remarkable interactions of CRGI with tumor immunology (**Figure 6N**).

### The high- and low-CRGI groups possessed different chemosensitivity

**Figure 7:**
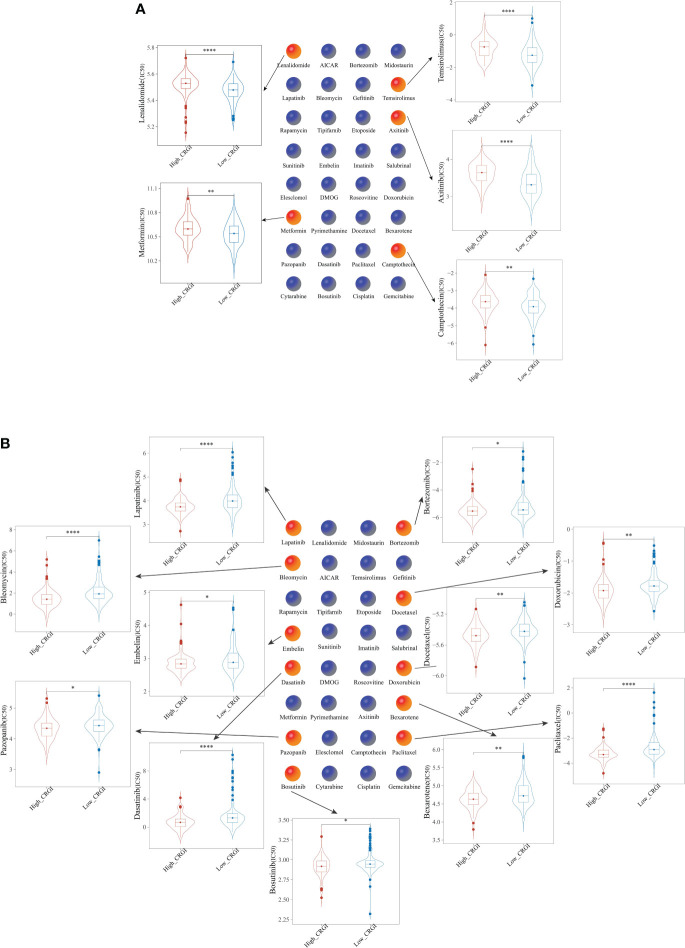
Comparison of the efficacy of 32 chemotherapeutics in **(A)** high-CRGI group, and **(B)** low-CRGI group. *, **, ***, and **** indicate the P-value thresholds 0.05, 0.01, 0.001, and 0.0001, respectively.

Chemotherapy has been the centerpiece in the treatment of cancer over the past few decades, yet due to the heterogeneous characteristics of tumors, even the responses to the same chemotherapeutic may vary from one patient to another (63). To address this problem, genome-based methodologies must be introduced. For this purpose, we evaluated the chemosensitivity of PAAD patients from the TCGA dataset who were classified into the high- and low-CRGI groups previously in the present study to 32 commonly used anticancer drugs (**Supplementary S3**). Subsequently, the half-maximal inhibitory concentration (IC50) value of 5 drugs (i.e., Lenalidomide, Metformin, Temsirolimus, Axitinib, and Camptothecin) was found relatively higher in the high-CRGI group (**Figure 7A**), while that of the other 11 drugs (i.e., Paclitaxel, Lapatinib, Dasatinib, Bleomycin, Docetaxel, Doxorubicin, Bexarotene, Gefitinib, Bosutinib, and Bortezomib) was found relatively higher in the low-CRGI group (**Figure 7B**).

### CRGI-based molecular subtypes were characterized by different survival outcomes, immunotherapy efficacy, and chemosensitivity

Since the above analytics has revealed that the high- and low-CRGI groups possessed distinct OS probability and immunotherapy efficacy, it raised our interest in systemically dividing it into more precise molecular subtypes through an unsupervised consensus method (i.e., K-mean algorithm). It was found that when K = 4, the PAAD samples were separated into 4 clusters in the consensus diagram (**Supplementary S4A**). Meanwhile, when K = 4, CDF almost reached its maximum which indicated good stability (**Supplementary S4B**). Besides, it was observed that CDF changed only slightly when K+/-1 (**Supplementary S4C**). Therefore, K = 4 was deemed to be an ideal value in the present study. To ensure the robustness of the clustering, we also conducted a principal component analysis (PCA), through which we could see that the samples were indeed well separated (**Supplementary S4D**). Therefore, ultimately, 4 molecular subtypes were identified, annotated by C1 (N = 37), C2 (N = 90), C3 (N = 23), and C4 (N = 29).

Furthermore, we examined the expression profiles of the 13 CRGI genes in each molecular subtype and found that generally, the expression levels were arranged in such order: C1 > C4 > C2 > C3 (**Supplementary S4E**).

We then investigated the clinical outcomes in these molecular subtypes. Results of the survival analysis suggested that C2 had the best prognosis, followed by C4, C3, and C1 (**Supplementary S4F**).

We also inspected the TIDE score and the expression of the representative genes of the soundest immune checkpoints in different molecular subtypes. For the TIDE score, there were differences with statistical significance between C1 and C3, and between C2 and C3, where C3 had a relatively lower TIDE score compared with that of C1 and C2 (**Supplementary S4G**). For the expression of the representative genes, except SIGLEC15, the rest were all found statistically significant and exhibited prominent differences in each molecular subtype (**Supplementary S4H**). In short, the 4 molecular subtypes demonstrated diverse immunogenic features, and that may lead to various efficacy in immunotherapy.

Finally, we carried out the elucidation of the chemosensitivity of PAAD patients from the TCGA dataset who were divided into 4 molecular subtypes based on the CRGI. This was done in the same way as we evaluated the chemosensitivity of the high- and low-CRGI groups. As a result, we identified that 27 of the 32 common anticancer medications exhibited changes that were statistically significant in each of the 4 molecular subtypes (**Supplementary S5**). Axitinib stood up as being particularly unique as it had exceptionally high statistical significance in its differences concerning both of the molecular subtypes.

### DLAT, LIPT1, and LIAS as reliable diagnostic biomarkers in PAAD

We selected a normal human pancreatic ductal epithelial cell line (i.e., HPDE6-C7) and 4 pancreatic cancer cell lines (i.e., CFPAC-1, PANC-1, SW1990, and AsPC-1) to examine the expression of *DLAT, LIPT1*, and *LIAS*.

**Figure 8:**
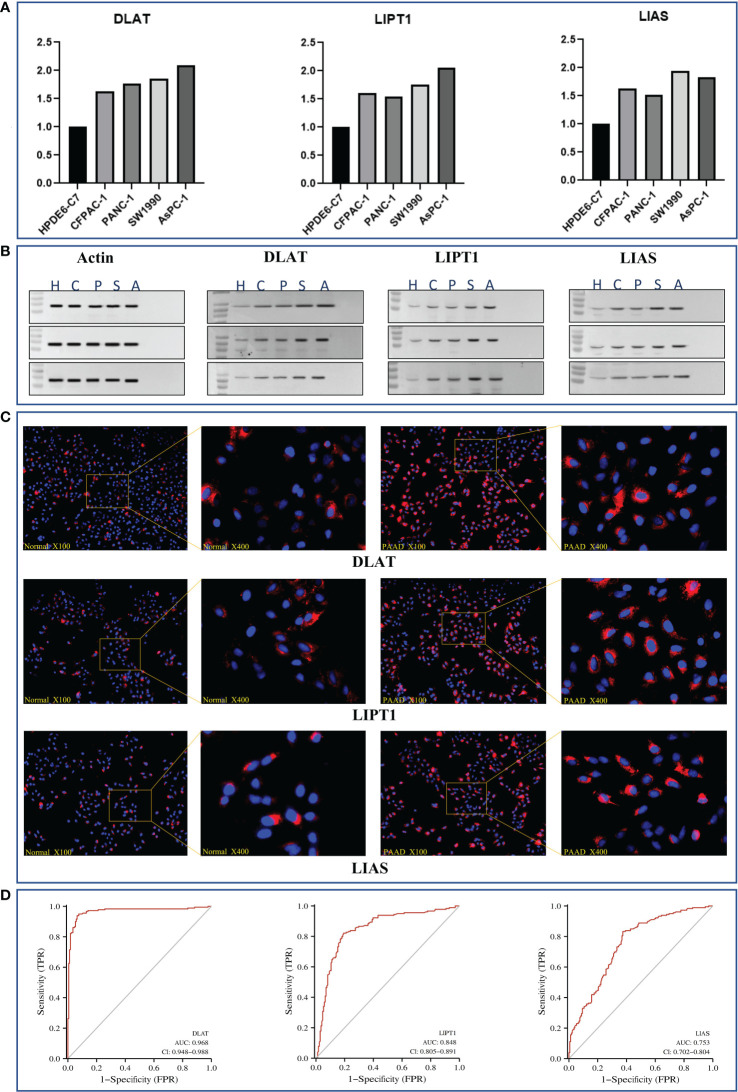
*In-vitro* validation of essential genes (i.e., *DLAT, LIPT1*, and *LIAS*) as potential diagnostic biomarkers in PAAD. **(A)** Results of RT-qPCR of *DLAT, LIPT1*, and *LIAS* in HPDE6-C7, CFPAC-1, PANC-1, SW1990, and AsPC-1 cell lines, respectively. **(B)** Results of western-blot in triple repletion utilizing the aforementioned cell lines. **(C)** IFS slides in 100X and 400X magnification demonstrated the expressional abundance of these proteins of interest in HPDE6-C7 and AsPC-1 cell lines. **(D)** Evaluation of the eligibilities of *DLAT, LIPT1*, and *LIAS* serving as diagnostic biomarkers in PAAD.

To be more detailed, we first performed a real-time quantitative polymerase chain reaction (RT-qPCR) to verify their expression at the upstream transcriptomic level. It was recognized that they were all expressed at a relatively higher level in the cancer cell lines than that in the normal ones (**Figure 8A**).

On the other hand, at the downstream proteomic level, we conducted a triple repetition in western blot to validate the expression of the corresponding proteins in the aforementioned cell lines. The results indicated that these proteins were expressed relatively higher in the cancer cell lines (**Figure 8B**; **Supplementary S6**). Macroscopically, we visualized these findings through immunofluorescence staining (IFS) using the HPDE6-C7 (i.e., normal cell line, control group) and AsPC-1 (cancer cell line, group of interest) cell lines. It was observed that the staining intensities were much higher in AsPC-1 than in HPDE6-C7 (**Figure 8C**).

The aforementioned results were further theoretically verified by utilizing the TCGA dataset through diagnostic ROC analysis. Consequently, we found *DLAT* demonstrated the highest accuracy with an AUC value that reached 0.968, followed by *LIPT1* with an AUC value of 0.848, and *LIAS* with an AUC value of 0.753 (**Figure 8D**).

## Discussion

Despite the fast growth of current healthcare technology, there has been very little progress in treating pancreatic adenocarcinoma (PAAD). The long-term overall survival (OS) rates of PAAD remain a major challenge for clinicians because of its high malignancy, rapid progression, and lack of effective treatments (14, 64). Less than 10% of the patients may survive for more than 5 years even until today (13–15). Under such circumstances, surgery and chemotherapy continue to be the most common treatments for PAAD. However, since these interventions lead to high morbidity and mortality, an expansion of the arsenal of translational medicine remains on-demand. In a word, the present situation is not optimistic. Although in clinical observation and animal modeling, a few validated biomarkers such as *KRAS, TP53, SMAD4*, and *CDKN2A* were discovered to exhibit apparent impacts on tumorigenesis, metastasis, and concomitantly poor prognosis (59), they were still regarded to be inadequate to cause the disease and less gratifying to be exploited in targeted drug development.

The good news is that as medicine has evolved from empirical to evidence-based, or even further, to personalized, the value of multi-omics data has raised unprecedented attention. Such rapid acquisition, storage, and analytics of such data in the era of big data make precision medicine more and more realistic. Under this background, bioinformatics as a subject within the intersection of computer science and biomedicine has made extensive progress in the past few decades. Subsequently, lots of genetic signatures and corresponding risk models were mined from internationally available genomic databases and increasingly accepted by the scientific community. In the past 10 years, newly characterized cell death processes like autophagy, ferroptosis, and pyroptosis are anticipated to provide new insights into the genesis and treatment of cancer including PAAD. At this point, the novel cell death mechanism termed “Cuproptosis” was reported by Tsvetkov et al. in March 2022 (2), lightening up the road for scientists, clinicians, and patients against PAAD.

Indeed, as copper serves as an essential trace element in the human body, its imbalance is tightly associated with numerous pathological conditions including cancers, albeit mainly through undefined underlying mechanisms. Dating back to the year 2013, Seiko et al. had proven that bioavailable copper was able to modulate the oxidative phosphorylation and growth of tumor tissues (65).

On the other hand, considering the complexity of tumor biology, it has been realized that it is insufficient to predict the clinical outcomes solely based on clinical and pathological characteristics or a single biomarker. Therefore, accurate assessment of the OS rate in PAAD has been an emerging concern.

Taking it altogether, in the present study, we optimized a Cuproptosis-gene index (CRGI) with important implications for prognosis, tumor immunology, molecular subtypes, and the efficacy of immunotherapy and chemotherapy through 10 mainstream algorithms in machine learning. Supplementarily, 3 essential CRGI genes (i.e., *DLAT, LIPT1*, and *LIAS*) were found with promising potential as diagnostic biomarkers through a computational method analyzing open datasets combined with *in-vitro* validation.

13 genes, including well-established biomarkers such as *KRAS, TP53, SMAD4, BRCA1, BRCA2*, and *CDKN2A* (17–20, 59, 66–69), as well as Cuproptosis-related genes such as *DLAT, LIPT1, LIAS, DLD, PDHA1, MTF1*, and *GLS40* comprises the CRGI. Among them, *CDKN2A*, a tumor suppressor gene that encodes for *p16INK4A* and *p14ARF*, critical for the regulation of cell cycle pathways (70, 71), was also a well-known biomarker in PAAD and a member of the 10 Cuproptosis-related genes meanwhile. The rest of the CRGI genes were indispensable to the process of mitochondrial energy production. For instance, *DLAT* was a critical component of the pyruvate dehydrogenase complex and was intimately engaged in the oligomerization of lipoylated TCA cycle proteins when copper ions are overloaded (72).

In comparison to other signatures derived from autophagy, ferroptosis, and pyroptosis, our model demonstrated a more robust and accurate predictive performance. Notably, such a conclusion was not only drawn from conventional assessment (i.e., the AUC value in the time-dependent ROC analysis) but also from the results of decision curve analysis (DCA), since we comprehended the necessity to maximize the goodness in clinical practice when tolerable false positivity and false negatives were inevitable (32).

Additionally, we investigated the relationship between the CRGI and tumor immunology and the differences in tumor immunology between the CRGI. We found that CRGI was associated with the condition of the tumor microenvironment, the cancer-immunity cycle, and immunotherapy efficacy. Regarding immunotherapy, in particular, the lower CRGI was more favorable as it indicated a substantially stronger response to immune checkpoint blockade and was more engaged with the cancer-immunity cycle and the 19 known biological processes in pancreatic cancer (46, 47).

The half-maximal inhibitory concentration (IC50) curves of 32 frequently used anticancer medicines were examined to determine their predicted chemotherapeutic effectiveness. Among them, the IC50 value of Lenalidomide, Metformin, Temsirolimus, Axitinib, and Camptothecin was found relatively higher in the high-CRGI group, while that of Paclitaxel, Lapatinib, Dasatinib, Bleomycin, Docetaxel, Doxorubicin, Bexarotene, Gefitinib, Bosutinib, and Bortezomib was found relatively higher in the low-CRGI groups. Both results came with statistical significance after log-rank testing.

Thereby, it is considered that the CRGI not only predicted the OS rate but also implicated the various therapeutic approaches that should be utilized for more precise therapy. As a further step, we aimed to use CRGI as a classifier for molecular subtype classification. Subsequently, it was found that immunotherapy and chemotherapy had distinct impacts on various molecular subtypes.

As the current diagnosis approach is underdeveloped and often shows delays in early detection (i.e., once the patient is identified as a PAAD patient, it is almost at a late stage of disease progression accompanied by metastasis to multiple organs), we hoped that the CRGI genes could help improve the situation to a certain extent. As such, *DLAT, LIPT1*, and *LIAS* were designated for further *in-vitro* research as they were more relatively weighted among the CRGI genes and relatively less studied previously. The results of both transcriptomic (RT-qPCR) and proteomic (western blot and immunofluorescent staining) assays suggested they were promising diagnostic biomarkers. The AUC value of each gene in the diagnostic ROC analysis using the TCGA dataset further verified the experimental phenomenon.

In summary, despite the imperfections, including the lack of real-world clinical cohort and own IC50 testing data, the present study highlighted the outstanding achievement of CRGI in PAAD prognostic prediction and the association with tumor immunology. These findings may inspire more and more immunotherapy- and chemotherapy-based interventions in the future.

## Data availability statement

The original contributions presented in the study are included in the article and **Supplementary Material**. Further inquiries can be directed to the first author.

## Author contributions

Conception and design: XH and SZ. Collection and assembly of data: XH. Data analysis and visualization: SZ, XH, AH, and JT. Data interpretation: XH and SZ. Manuscript writing: XH and SZ. Manuscript revision: all authors. Final approval of the manuscript: all authors.

## Funding

This work is funded in part by the project TKP2021-NKTA-34, implemented with the support provided by the National Research, Development and Innovation Fund of Hungary under the TKP2021-NKTA funding scheme.

## Acknowledgments

All the aforementioned *in-vitro* experiments (i.e., cell culture, RT-qPCR, western blot, and IFS) were performed by Chengdu Guichuang Biotechnology Ltd and Shanghai Hanwan Biotechnology Development Center. We deeply thank them for their technical support in the present study.

## Conflict of interest

The authors declare that the research was conducted in the absence of any commercial or financial relationships that could be construed as a potential conflict of interest.

## Publisher’s note

All claims expressed in this article are solely those of the authors and do not necessarily represent those of their affiliated organizations, or those of the publisher, the editors and the reviewers. Any product that may be evaluated in this article, or claim that may be made by its manufacturer, is not guaranteed or endorsed by the publisher.

## Supplementary material

Supplementary S1 Correlation between the CRGI and clinical factors.

Supplementary S2 Submap validation.

Supplementary S3 Comparison of the efficacy of 32 anticancer drugs in the high- and low-CRGI groups.

Supplementary S4 Characterization of 4 CRGI-based molecular subtypes with different survival outcomes and immunotherapy efficacy.

Supplementary S5 Comparison of the efficacy of 32 chemotherapeutics across the CRGI-based molecular subtypes.

Supplementary S6 Western blot original result.
