# Supplementary material for: Cuproptosis-related gene index: A predictor for pancreatic cancer prognosis, immunotherapy efficacy, and chemosensitivity
Source: Front Immunol. 2022 Aug 25;13:978865. doi: 10.3389/fimmu.2022.978865 (PMC9453428; doi:10.3389/fimmu.2022.978865)
Supplement: Supplementary file 1 [file Table_1.pdf]

Target Name	Primer	
Actin	F	TCCTCCTGAGCGCAAGTACTCC
	R	CATACTCCTGCTTGCTGATCCAC
LIAS	F	CTGAACACATTGCAAAGACCG
	R	CATGTTTCAGTACACGTAGGGAC
LIPT1	F	CATATGAATCTAGAAGGCAAACC
	R	TCCTCCACTTCTTCTCCGA
DLAT	F	ACTCCCCAGCCTTTAGCTC
	R	CAATCCCTTTCTCTACTGCCAAC

T1_Primer sequences for RT-qPCR.
