# Supplementary material for: Cuproptosis-related gene index: A predictor for pancreatic cancer prognosis, immunotherapy efficacy, and chemosensitivity
Source: Front Immunol. 2022 Aug 25;13:978865. doi: 10.3389/fimmu.2022.978865 (PMC9453428; doi:10.3389/fimmu.2022.978865)
Supplement: Supplementary file 2 [file Table_2.pdf]

Target Name	Antibody Name	Antibody Molecular Weight	Source Company
Actin	[mAbcam 8226] - (ab8226)	43kDa	Abcam
LIAS	(ab246917)	42kDa	Abcam
LIPT1	(bs-18298R)	38kDa	Bioss Antibody
DLAT	(4A4-B6-C10) Mouse mAb #12362	63kDa	CST

T2_Antibody information
