# Supplementary material for: Cuproptosis-related gene index: A predictor for pancreatic cancer prognosis, immunotherapy efficacy, and chemosensitivity
Source: Front Immunol. 2022 Aug 25;13:978865. doi: 10.3389/fimmu.2022.978865 (PMC9453428; doi:10.3389/fimmu.2022.978865)
Supplement: Supplementary S1 — Correlation between the CRGI and clinical factors. [file Image_1.pdf]

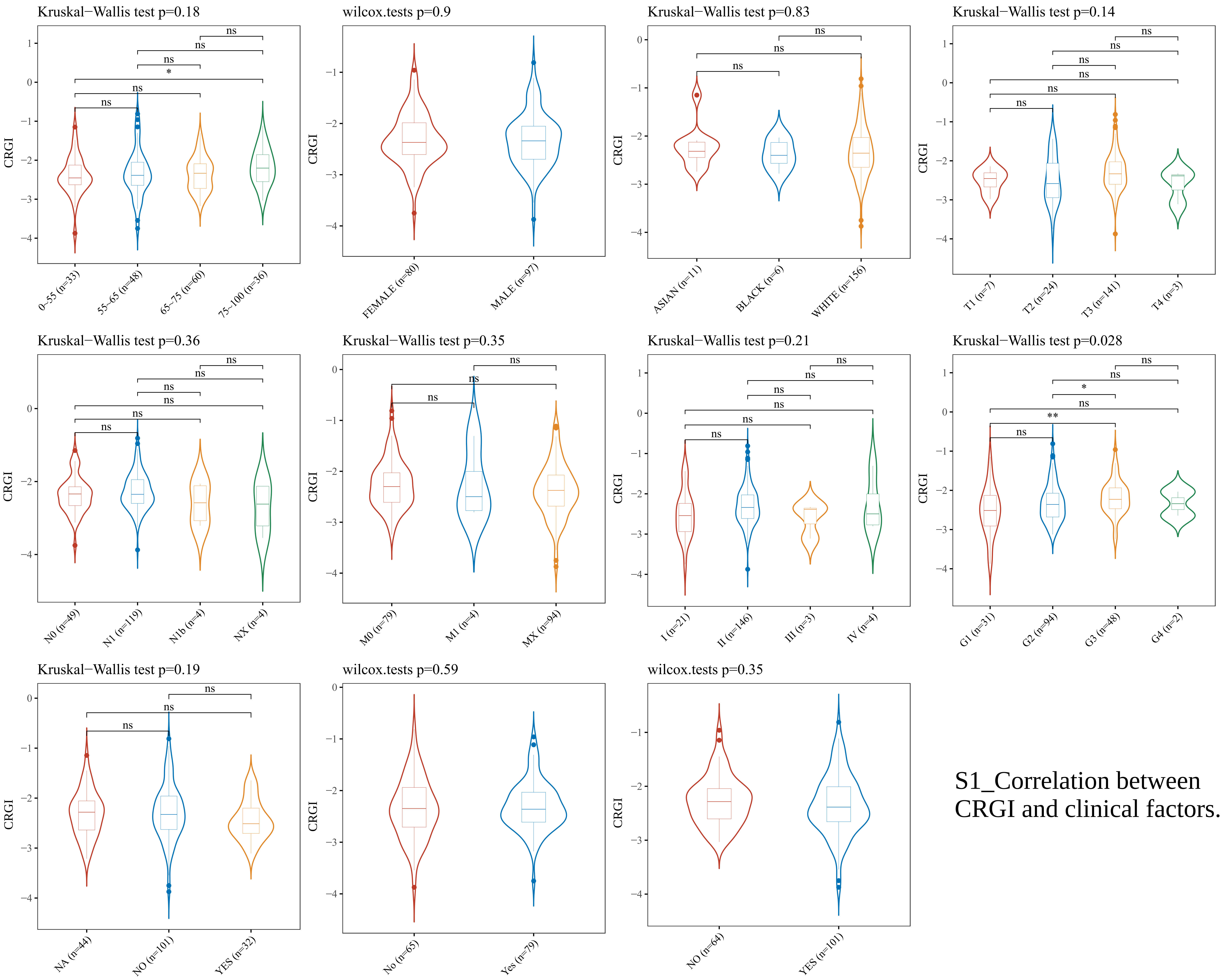

S1_Correlation between CRGI and clinical factors.
