# Supplementary figures and images for: Cuproptosis-related gene index: A predictor for pancreatic cancer prognosis, immunotherapy efficacy, and chemosensitivity
Source: Front Immunol. 2022 Aug 25;13:978865. doi: 10.3389/fimmu.2022.978865 (PMC9453428; doi:10.3389/fimmu.2022.978865)

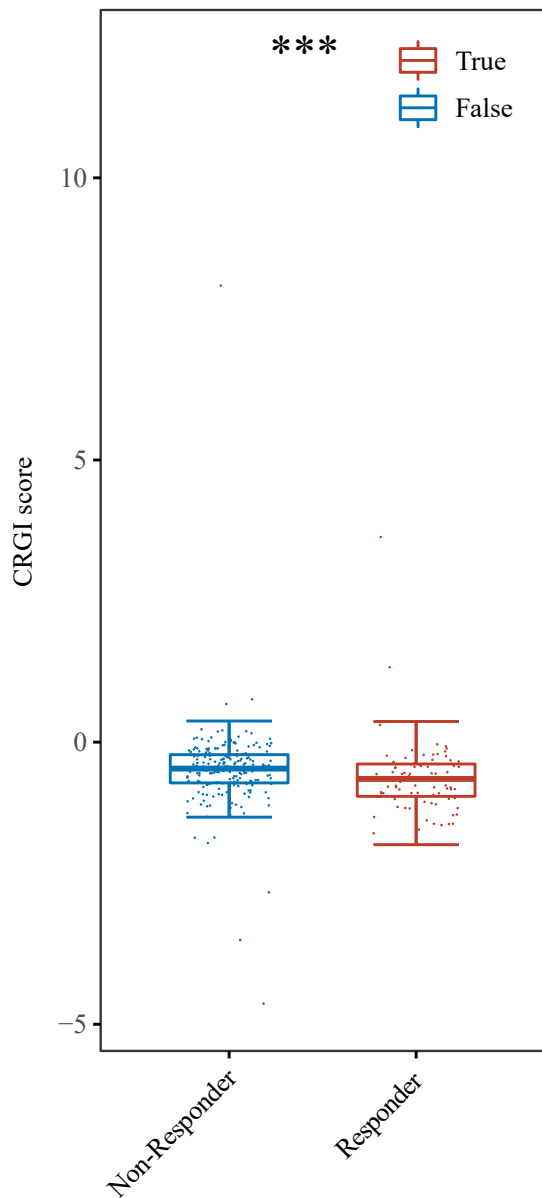

**A**

S2\_Submap validation

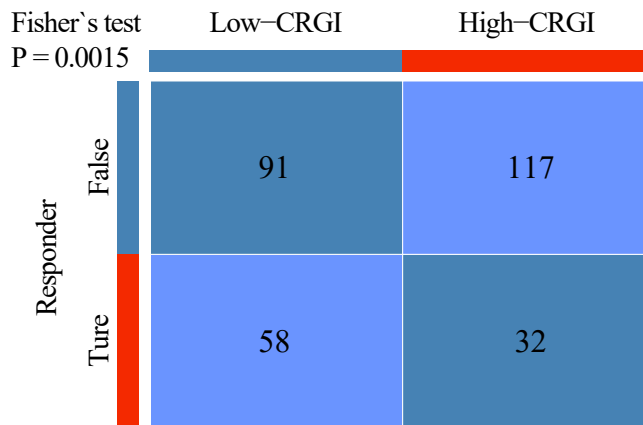

**B**

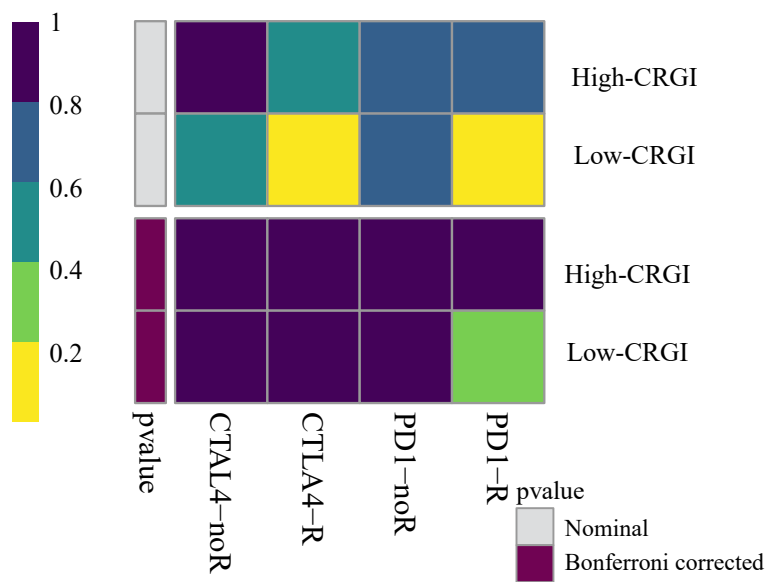

**C**

Supplement: Supplementary S2 — Submap validation. [file Image_2.pdf]

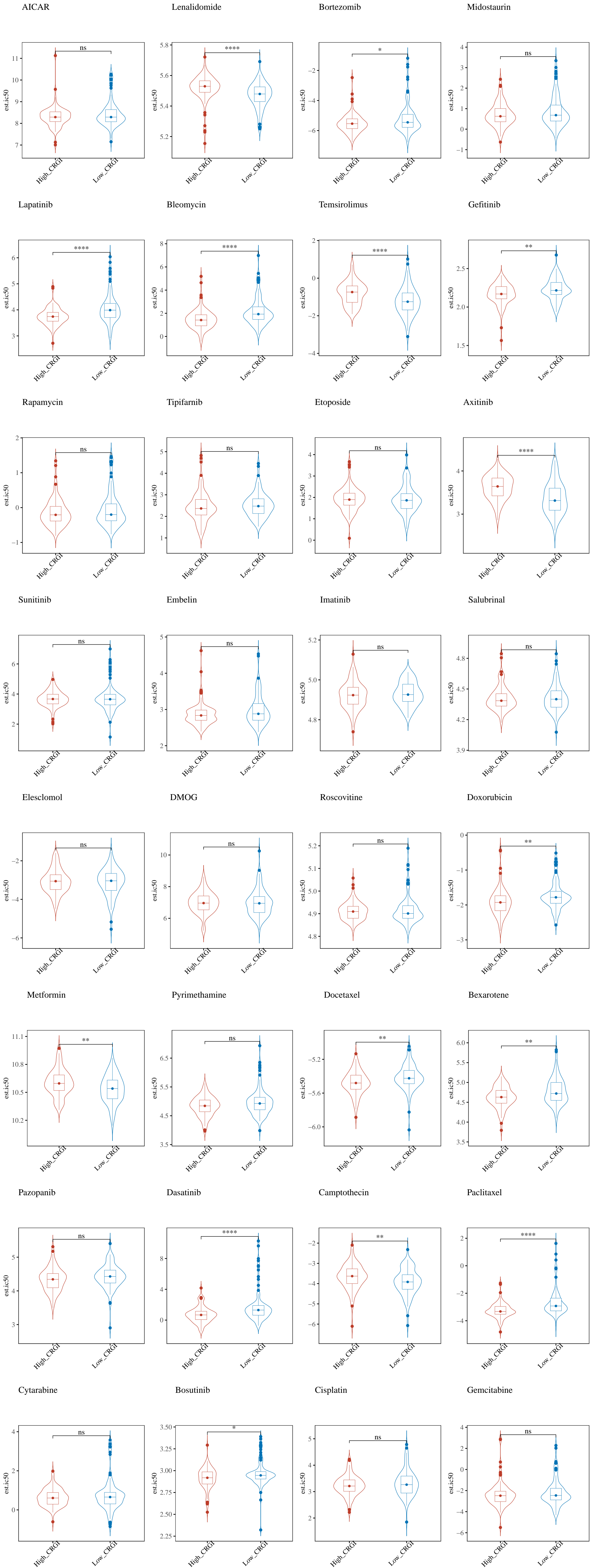

S3\_Comparison of the efficacy of 32 anti-cancer drugs in high- and low-CRGI groups.

Supplement: Supplementary S3 — Comparison of the efficacy of 32 anticancer drugs in the high- and low-CRGI groups. [file Image_3.pdf]

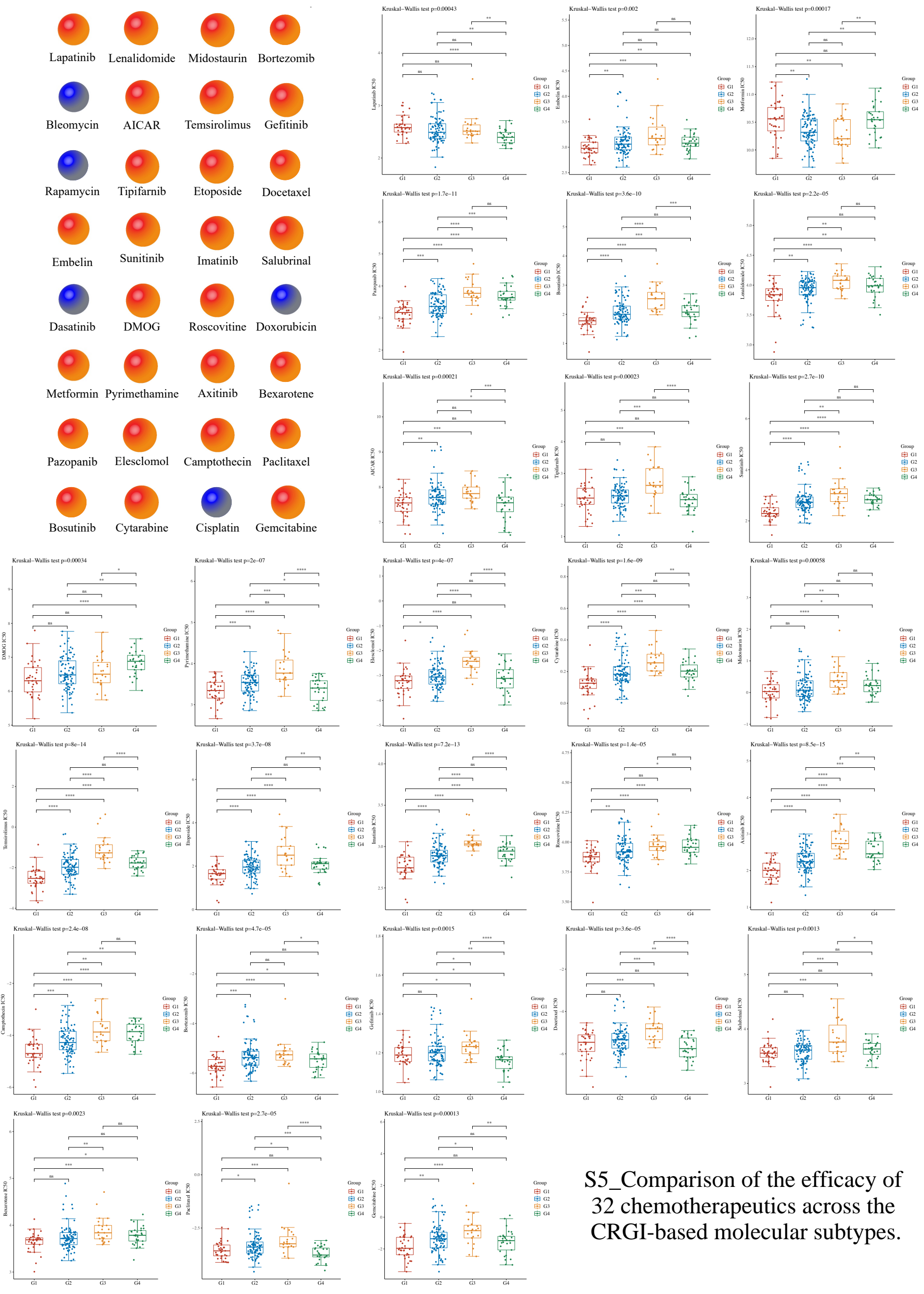

Supplement: Supplementary S5 — Comparison of the efficacy of 32 chemotherapeutics across the CRGI-based molecular subtypes. [file Image_5.pdf]

Actin

DLAT

LIPT1

LIAS

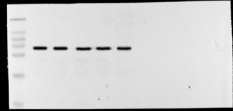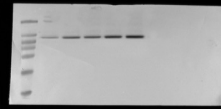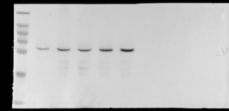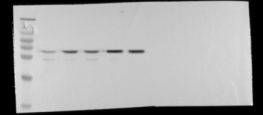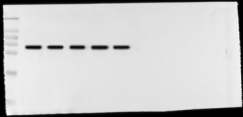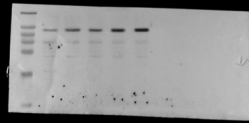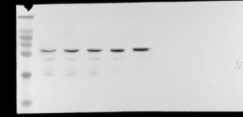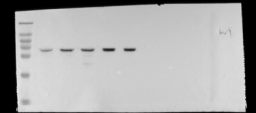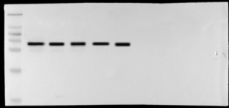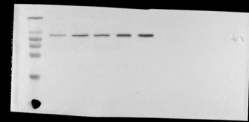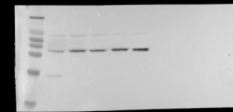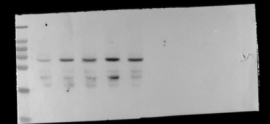

S6\_Western blot original result

Supplement: Supplementary S6 — Western blot original result. [file Image_6.pdf]
