# Supplementary material for: Cuproptosis-related gene index: A predictor for pancreatic cancer prognosis, immunotherapy efficacy, and chemosensitivity
Source: Front Immunol. 2022 Aug 25;13:978865. doi: 10.3389/fimmu.2022.978865 (PMC9453428; doi:10.3389/fimmu.2022.978865)
Supplement: Supplementary S4 — Characterization of 4 CRGI-based molecular subtypes with different survival outcomes and immunotherapy efficacy. [file Image_4.pdf]

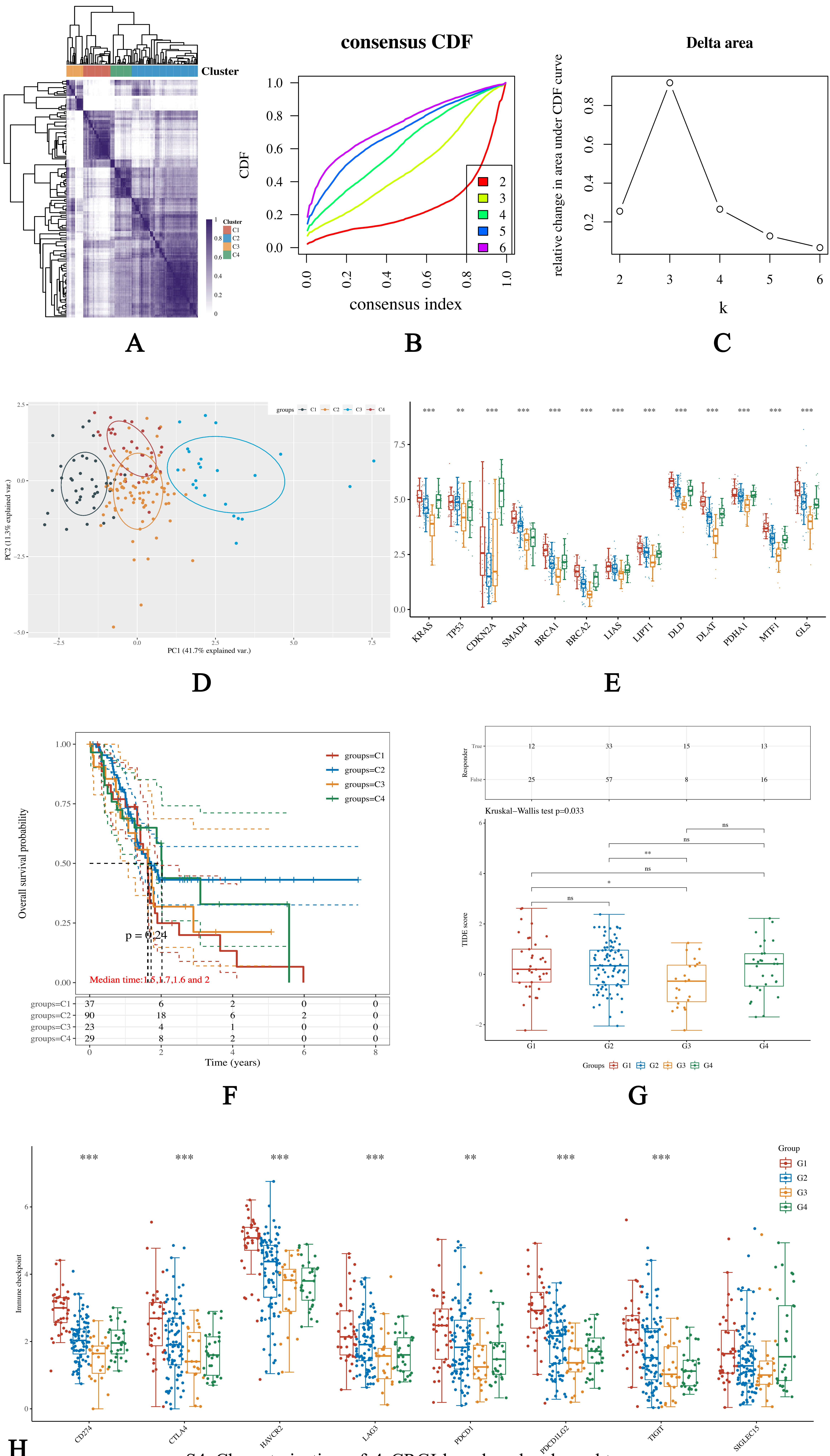

S4_Characterization of 4 CRGI-based molecular subtype withdifferent survival outcomes and immunotherapy efficacy.
